# Quantiferon Gold-in-tube assay for TB screening in HIV infected children: influence of quantitative values

**DOI:** 10.1186/1471-2334-14-516

**Published:** 2014-09-23

**Authors:** Winsley Rose, Ian Kitai, Fatima Kakkar, Stanley E Read, Marcel A Behr, Ari Bitnun

**Affiliations:** Department of Pediatrics, Christian Medical College, Vellore, 632004 India; Department of Pediatrics, Hospital for Sick Children, University of Toronto, Toronto, Canada; University of Montreal, Montreal, Canada; McGill International TB centre, Montreal, Canada

**Keywords:** HIV, Tuberculosis, Screening, IGRA, Quantiferon

## Abstract

**Background:**

HIV infected children are at increased risk of TB disease and require annual TB screening. Data on use of IGRA for TB screening in them are limited. We retrospectively evaluated the usefulness of Quantiferon Gold-in-tube test (QFT), an IGRA in screening for LTBI in relatively healthy, immunologically stable HIV infected children.

**Methods:**

HIV infected children with no prior history of TB were screened for latent TB as part of routine care. They underwent risk of TB assessment, TST and QFT. QFT was repeated twice or three times depending on the quantitative values. Independent test validation was also performed.

**Results:**

Eighty one children had 109 QFT tests. All had adequate mitogen responses. The initial QFT was positive in 15 (18.5%) children; quantitative IGRA responses were 0.35-1.0 IU/mL in 9 (60%), 1.0-10 IU/mL in5 (33.3%) and >10 IU/mL in 1 (6.7%). None that tested positive had documented TB exposure or TB disease. Baseline characteristics in the QFT positive and negative groups were similar. Repeat testing within 17 weeks demonstrated reversion to negative in 79% of cases. Repeat blinded independent testing of all QFT positive results and a random selection of initial negative tests demonstrated concordance in 96% of cases. Seven children (QFT > 1.0 IU/mL or positive TST) were offered INH preventive therapy. In no case has TB disease developed in 2 years of close follow-up.

**Conclusions:**

QFT is a valid method for LTBI screening relatively healthy, immunologically stable HIV infected children. However, reversion to negative on repeat testing and lack of correlation with TST results and risk of TB exposure makes interpretation difficult.

**Electronic supplementary material:**

The online version of this article (doi:10.1186/1471-2334-14-516) contains supplementary material, which is available to authorized users.

## Background

Tuberculosis is the leading cause of death in persons living with HIV. It is estimated that one third of the 33.3 million people living with HIV worldwide are infected with tuberculosis (TB). The annual risk of developing TB disease in this context is between 7% and 10% [1]. Moreover, overall mortality is twofold higher for HIV/TB co-infected individuals compared to those with isolated HIV infection [2]. Consequently, diagnosis of latent TB infection (LTBI) and provision of chemotherapy to those testing positive for LTBI is strongly recommended [3].

The tuberculin skin test (TST) has been used for decades to diagnose LTBI [4]. For HIV infected children an annual TST is recommended due to their high risk of progression to disease [3]. However, the utility of the TST is limited due to suboptimal sensitivity and specificity [5]. False negative TST results are of particular concern [6].

Interferon gamma release assays (IGRAs) are now recommended as an alternative method of diagnosis of LTBI in HIV infected persons [5]. IGRAs have performed well in HIV infected adults without severe immune suppression, but less so in those with severe immune compromise, leading to large numbers of indeterminate response [7, 8]. However, there is a paucity of data on the utility of IGRA in HIV infected children especially in low TB burden setting. In high TB endemic setting, poor to moderate agreement between TST and IGRAs have been reported in both HIV infected adults and children [9]. Recent data from South Africa, a high TB burden country showed that IGRAs were more likely to be positive in HIV uninfected compared to HIV infected children [10]. In this report, we present our experience on the use of the Quantiferon Gold-in-tube test (QFT) in screening for LTBI in a relatively healthy, immunologically stable, cohort of HIV infected children in a low incidence setting.

## Methods

We retrospectively reviewed the results of screening for LTBI performed in our HIV clinic over a one year period (2010–2011). During this period QFT had been used on a trial basis concurrent with TST as a matter of routine. All HIV-infected children less than 19 years of age attending the pediatric HIV clinic at the Hospital for Sick Children, Toronto, with no prior history of TB infection or disease were screened for latent TB infection as part of routine care. A detailed questionnaire was administered to all children and/or their caregivers, as appropriate, to assess their risk for exposure to TB. BCG vaccine status was assessed by history and the presence of BCG scar. All those who had a history of BCG vaccination or the presence of a BCG scar were considered to have had BCG vaccine. A TB incidence of >25/100,000 population was considered to be a TB endemic country. All children underwent complete physical examination and blood testing including viral load and CD4 count in accordance with routine clinical care. All children underwent TST and QFT testing on the same day. The study was approved by the Research Ethics Board of the Hospital for Sick Children.

Tuberculin skin testing was performed by the Mantoux method using TUBERSOL® (Tuberculin Purified Protein Derivative, Sanofi Pasteur). TST was considered positive if the induration at its widest transverse diameter was greater than or equal to 5 mm 48–72 hours after placement in accordance with the Canadian Tuberculosis Standards [11]. The results of the first TST were read by a physician or other appropriately trained health care professional where possible and the rest were self read by the child’s caregiver. The caregivers were provided with a tuberculin test record form that contains palpable examples of 0 mm, 1–7 mm, 7–9 mm and ≥10 mm induration. They were orally instructed to feel the child’s TST site between 48 and 72 hours after placement and to immediately see a physician if they noticed any swelling or induration in order to confirm the induration and measure its size. For those whose initial TST was self-read and had an initial positive QFT, the TST was repeated and read by a physician or other appropriately trained health care professional when they came for their routine clinical follow up visit.

QFT assay was performed using the QuantiFERON®-TB Gold In-Tube (Cellestis) test according to manufacturer instructions [12]. Blood was drawn by the on-site pediatric phlebotomy service. The tubes were shaken gently to coat the sides of the tubes and transported to an on-site laboratory within 2 hours. The tubes were then incubated for 16–24 hours, centrifuged and the plasma was removed and frozen. The QFT assay was done in batches of 26 samples. For each batch an 8 point standard curve was obtained according to manufacturer’s instructions and quality control criteria and using manufacturer supplied reagents. Results were interpreted according to manufacturer’s guidelines: an IFN concentration of 0.35 IU/ml over the nil concentration was classified as positive. Nil concentrations of 8.0 IU/ml and mitogen differences of 0.5 IU/ml were considered indeterminate. The laboratory performing the assay participates in an ongoing general quality control program.

All children with an initial positive QFT or TST were re-evaluated clinically and with chest radiography to look for signs of active TB. The QFT was repeated in all such children. For those who did not have a consistent QFT response (TB antigen response corrected for Nil control) > 1.0 IU/mL in the second testing, QFT was repeated for the third time. All initial and repeat positive QFT tests (>0.35 IU/mL) as well as 8 randomly selected negative tests were repeated in another laboratory with expertise in performing the test in a blinded manner from the original batched frozen samples to validate test results.

## Results

Between July 2010 and June 2011, all 83 HIV infected children attending the clinic were screened for latent TB. Eighty one were included in our analysis; 2 children previously treated for pulmonary TB were excluded. The mean age of participants was 12.5 ± 4.3 years, 43(55%) were female and 6 (7%) were less than 5 years of age. None had a documented history of TB exposure. Approximately 30% of the children (Africa 19%, South and Central America 5%, South and South East Asia 4%, Russia 1%, Europe 1%) and 86% of their mothers (Africa 61%, South and Central America 1%South and South East Asia 7%, Europe 17%) were born in countries endemic for TB, and 32% of the children had received BCG vaccine prior to arrival in Canada. According to the revised CDC HIV criteria, at the time of presentation, 9 subjects (11%) were classified as having had severe symptoms (class C), 21 subjects (26%) as having had moderate symptoms (class B), 28 subjects (35%) as having had mild symptoms (class A) and 23 subjects (28%) as having had no symptoms (class N). With regards to the CDC immunological categories, a history of severe immune suppression, moderate immune suppression, and no suppression was noted for 25(30%), 28 (35%) and 28 (35%) subjects, respectively. Mean CD4 percent, and total CD4 count at the time of QFT testing were 31.9% ± 9.6%, and 827 ± 459 cells/mL, respectively. Sixty eight (82%) were antiretroviral experienced, although only 64 subjects (79%) were currently receiving combination ART.

Baseline demographic and HIV-related characteristics are depicted in Table 1. There were no significant differences between groups with respect to age, sex, body mass index, place of birth, place of mother’s birth, CDC clinical or immunologic category, receipt of antiretroviral therapy, CD4 percent, CD4 count, or viral load.

**Table 1:** **Baseline characteristics**

Characteristics	QFT + (n = 15)	QFT- (n = 65)	P
**Mean age**	13.8(+/−3.5)	12.1+/−4.5	0.16
**Age < 5**	0	6(9%)	0.22
**Females**	5(33.3%)	38(57.5%)	0.09
**Mean BMI**	20.6+/−3.6	20.2+/−4.6	0.71
**Born in TB endemic region**	10(66.7%)	46(69.7%)	0.82
**Mother born in TB endemic region**	14(93.3%)	56(84.9%)	0.39
**BCG vaccinated**	6(40%)	20(30.3%)	0.47
**Mean CD4%**	31.4+/−11.3	32.1+/−9.3	0.79
**CD4 > 25%**	10(66.7%)	53(80.3%)	0.51
**CD4 15-25%**	4(26.7%)	10(15.2%)	
**CD4 < 15%**	1(6.7%)	3(4.6%)	
**Mean CD4 count**	744+/−331.5	846.7+/−483.8	0.44
**Median CD4 count (IQR)**	769(481–1043)	731(525–1012)	0.88
**Viral load log10 copies/ml**	3.6+/−1.3	3.5+/−0.86	0.87
**Undetectable viral load**	10(66.7%)	39(59.1%)	0.59
No symptoms*	5(33.3%)	18(27.3%)	0.91
Mild symptoms*	5(33.3%0	23(34.8%)	
Moderate symptoms*	4(26.7%)	17(25.8%)	
Severe symptoms*	1(6.7%)	8(12.1%)	
**Proportion on ARV**	13(86.7%)	55(83.3%)	0.75
**Median duration of ARV in years (IQR)**	7.33(2.2-10.84)	8.04(1.84-11.8)	0.91
**3TC monotherapy**	0	4(6.1%)	0.73
**Combination ART**	13(86.7)	51(77.3%)	0.42

*at initial presentation; BMI – Body Mass Index; CD 4 – Cluster of Differentiation 4; ARV – Anti Retroviral Therapy; 3TC –Lamivudine.

**Figure 1:**
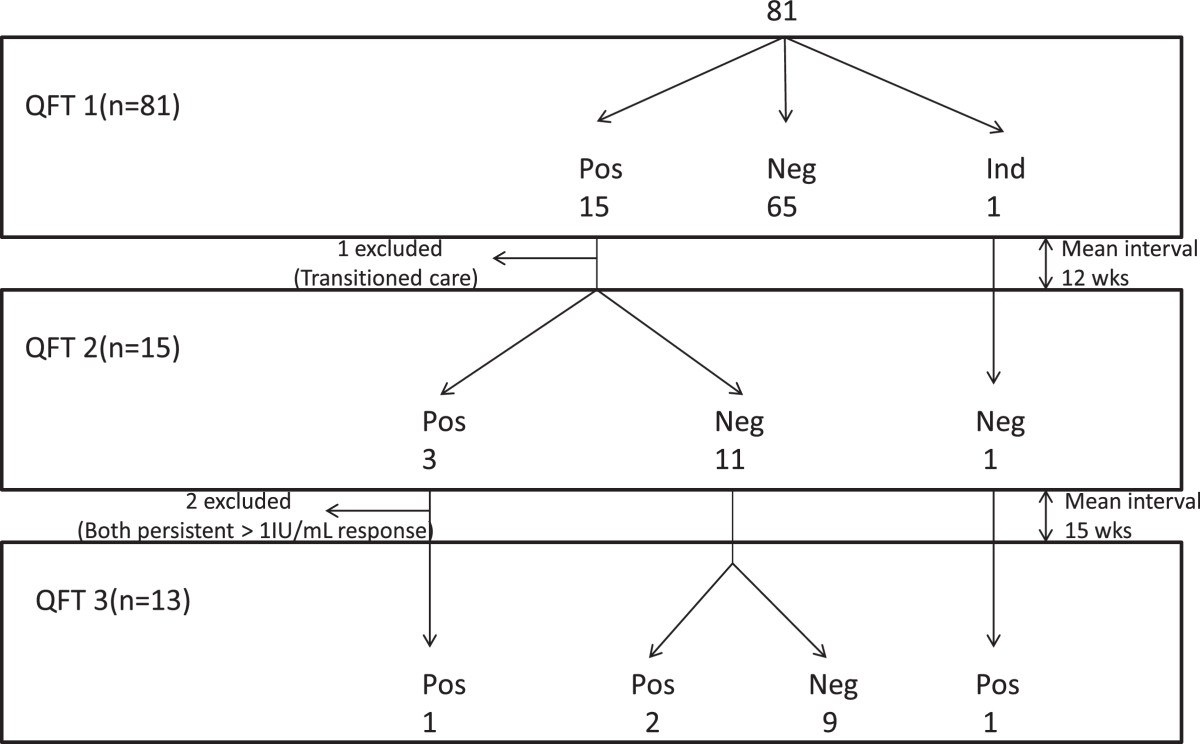
**Flowchart of QFT testing.**

The details of QFT testing over time are provided in Figure 1 and Table 2. In total, there were 109 tests performed on 81 children. All had adequate mitogen responses including 105 (96.3%) with values of > 10 IU/mL, the others being 8.5 IU/mL, 8.03 IU/mL, 6.54 IU/mL and 3.28 IU/mL. All 5 children whose CD4 count was less than 350 cells/μL (lowest being 120 cells/μL) had robust mitogen responses >10 IU/mL. Eighty of 81 children had valid initial QFT results; one had an indeterminate result due to high IFN-gamma activity in the nil control tube (>10 IU/mL). QFT testing was repeated twice on this patient, both with valid results.

**Table 2:** **TB antigen response (corrected for Nil control) in those with positive first QFT result**

	QFT −1 (IU/mL)	Repeat (IU/mL)	QFT −2 (IU/mL)	Repeat (IU/mL)	QFT −3 (IU/mL)	Repeat (IU/mL)	TST (mm)
1	3.71	9.68	5.41	5.52			0
2	2.88	4.1	5.28	5.27			0
3	>10	>10	0.31	0.38	−0.02	0.01	0
4	2.23	1.98	−0.03	−0.01	0.02	0	0
5	1.64	1.19	0.08	−0.03	0.08	0.09	0
6	1.07	1.21	0.92	0.94	0.42	0.25	0
7	0.92	1.16	0.29	0.21	0.38	0.37	0
8	0.7	0.9	0.03	0.28	0.03	0.06	0
9	0.53	0.53	0.23	0.14	0.8	0.87	0
10	0.51	0.42	0.04	0	0.02	0.09	0
11	0.43	0.76	0.14	0.11	−0.04	0	0
12	0.42	0.46	0.06	0.02	0.03	0	0
13	0.39	0.59	0.07	0	0.01	0.01	13
14	0.36	0.36	0.04	−0.02	−0.01	0.01	0
15	0.45	Transitioned to adult care (no repeat testing)					0

Positive QFT result > 0.35 IU/mL.

The initial QFT was positive in 15 children. Of these, 9(60%) were low positive with TB antigen responses (corrected for Nil control) between 0.35 IU/mL and 1.0 IU/mL (Table 2). Of the remaining 6 children, 5(33.3%) had TB antigen responses (corrected for Nil control) between 1.0 IU/mL and 10 IU/mL and 1 (6.7%%) had robust TB antigen response (corrected for Nil control) >10 IU/mL. There were no significant differences between the children who had their first QFT IT positive or negative with respect to age (p = 0.24), BMI (p = 0.38), country of birth (p = 0.63), country of mother’s birth (p = 0.34), CD4 count (p = 0.83) or viral load (p = 0.45). The two children excluded from this analysis because of prior treatment for TB both had positive QFT results with responses >10 IU/ml.

The QFT was repeated in 14 of the 15 children with initial positive results between 7 and 17 weeks (mean 12 weeks) after the first QFT; one was not repeated because the child had transitioned to adult care in the interim. None of the children had a change in their immunologic status or antiretroviral therapy between tests. Eleven (79%) of the 14 second QFT tests reverted to negative including all of those that had an initial low positive QFT of 1.0 IU/mL or less. Of the 5 children whose initial QFT value was above 1.0 IU/mL, 3 remained positive, 2 reverted to negative.

The 2 children who had repeated QFT values of greater than 1.0 IU/mL were placed on treatment for LTBI. The remaining 12 had a third QFT performed between 6 and 28 weeks (mean 14 weeks) after the second QFT assay. Of these, 9 remained negative, 2 reverted to low positive and one remained low positive (Table 2). The child whose initial QFT was indeterminate had his QFT IT repeated twice and both had valid results; the second QFT result was negative (0.86 IU/mL with high background IFN activity of 6.02 IU/mL in the nil tube), the third positive (2.38 IU/mL).

Forty eight samples from the original batched samples were retested in an alternate laboratory, of which 40 were initially positive and 8 were initially negative. Forty six out of the 48 (96%) samples had consistent interpretative results on retesting (Table 2). The repeat blinded testing of the initially negative results were selected using an online random number generator programme (http://www.random.org/integers/) using the study numbers. If the particular study number had an initial positive QFT result, that number was skipped. Eight such samples were randomly selected. The 8 randomly selected negative tests remained negative upon repeat testing in the second laboratory with a mean difference of 0.036 IU/mL (range 0 – 0.15 IU/mL). Of the 2 discrepant results one went from negative to positive (0.31 IU/mL to 0.38 IU/mL) and one reverted to negative (0.42 IU/mL to 0.25 IU/mL).

Of the 81 included children, 65 (80.2%) had initial self-read TST’s, the remainder being physician read. Of those with positive QFT’s, 10 were self-read and 5 were physician read. All children whose initial TST was self-read, had repeat physician read TST’s 97 to 350 days (mean 185 days) after the self-read TST. None had known contact with TB in the interval between tests. None of the TSTs done the first time were positive. Only one of the repeat tests was positive. In this child the physician read repeat TST demonstrated 13 mm of induration. The patient’s initial QFT was a low level positive (0.39 IU/mL) while 2 subsequent QFT’s were negative. He was an 11 year old boy, born in Burundi, who had moved to Canada 4.5 years prior to the reactive TST. He had no known TB contacts, was clinically well, and had a normal chest radiograph. He had received BCG vaccine in early infancy.

All the children with positive first QFT were evaluated clinically and radio graphically for signs of active TB and none of them had any symptoms or signs of disease. All children with TB antigen response (corrected for Nil control) >1 IU/mL or a positive TST were offered INH preventive therapy. Seven children received INH preventive therapy. In no case has TB disease developed in 2 years of close follow-up.

## Discussion

Our results indicate that QFT testing of relatively healthy, immunologically stable, HIV infected children produces valid results in the majority of cases. This would indicate that QFT can be used in this population. However, several potential problems were observed. First, only one A child with positive QFT results had positive TST result. Given that these children were relatively healthy and immunologically stable and had no history of TB exposure, one would expect strong concordance with TST. Second, as has been observed in other patient populations and in healthcare workers, there were significant reversions particularly in those with low positive results (0.35-1.0 IU/mL range) [13]. Thus, the reliability of the currently recommended interpretive cut-off for a positive QFT test should be questioned. Guidelines based on this cut off for HIV infected children [5] may need to be revised to incorporate quantitative IFN-gamma responses.

The unexpectedly high rate of positive QFT results was a surprising observation. A detailed clinical assessment of the children with positive results demonstrated that none had documented exposure to tuberculosis or clinical or radiographic evidence of TB disease, and none had reactive TSTs. Furthermore, repeat QFT testing within 17 weeks of the first test demonstrated reversion to negative in 11 of 14 cases. A review of phlebotomy and laboratory procedures found no issues related to sample collection, labelling or processing. Because of reports linking false positive QFT results to specific lots [14], we reviewed all QFT tests performed between September and November 2010, when our study samples were tested. In one run there was a 50% positive rate in 26 non-HIV infected patients, but these were all close contacts with positive TSTs. Testing of other patients through the laboratory also produced results that correlated very well with gradient of exposure [15]. Finally, the QFT results from 48 samples in our lab showed 96% concordance with those obtained in a blinded fashion by an independent laboratory. Taken together, these observations strongly suggest that test performance was excellent and the results valid from a quality assurance perspective, and raise serious doubts as to the currently-recommended interpretive cut-off for a positive QFT test.

The rate of indeterminate results is an important measure of the usefulness of the QFT. The observation that only 0.9% of the 109 tests done in our study produced indeterminate results is reassuring in this regard. This finding corroborates those of several other studies involving relatively healthy HIV infected adults. The rate of indeterminate results ranged from 2–6% in studies of HIV infected adults in high income countries [16–22]. By contrast, high rates of indeterminate results (20% and 66%) have been noted in children with immune compromising conditions other than HIV [23, 24] and in otherwise healthy children less than 5 years of age [25]. In our study, the one child with indeterminate result was 18 years old with a CD4 count of 585/μL and undetectable viral load.

Conversions and reversions have been observed with serial QFT testing making interpretation of a single QFT result difficult. In a study of healthy adults treated for active tuberculosis, reversion to negativity was observed in 32.1% of cases [9]. Among health care workers who underwent weekly testing, 28.6% displayed conversions and reversions, all with TB antigen responses (corrected for Nil control) <3.0 IU/mL [26]. Pai et al. reported 24% reversions on serial testing in health care workers with 78% of them occurring with TB minus nil responses <1.0 IU/ml [13]. Possible explanations for reversions include clearing of TB infection, biological variation in interferon gamma levels, or variability in laboratory and test procedures. There may be a zone of uncertainty around the manufacturer’s cut off of 0.35 IU/mL where reversions and conversions are more frequent [27].

In our study, serial testing was done only for those who had an initial positive QFT. Hence our data reflect more reversions than conversions upfront. Our rates of inconsistent response for those with positive QFT was 78.6% (11/14) among those tested on 2 occasions and 91.6% (11/12) for those tested 3 times. All 8 children with TB antigen response (corrected for Nil control) values of 0.35-1.0 IU/mL who had serial QFT testing done, had inconsistent responses on serial testing. These findings concur with those observed using the ELISpot test in which almost 50% of HIV-infected children with probable or definite TB disease demonstrated reversions during treatment and another 50% converted from negative to positive during treatment [28].

Many guidelines have been developed incorporating IGRAs in screening for LTBI. The latest United States Centers for Disease Control (CDC) recommendations advocate for the use of either an IGRA or TST in screening for LTBI, with a caveat that even multiple negative test results cannot fully exclude TB infection [5]. The latest Canadian guidelines adopt a step wise approach for HIV infected subjects whereby TST is recommended as an initial screen and the IGRA’s reserved as optional for those whose TST was negative [29]. Neither guideline makes a distinction between robust and borderline positive IGRA results. Our data, as well as those of others [13, 27], suggest that some allowance should be provided in the interpretation of low IFN-gamma responses (TB antigen response corrected for Nil control values 0.35-1.0 IU/mL) where reversions are particularly common. While further study is needed to fully understand the significance of these low positive results, it is quite plausible that many of these subjects do not have LTBI. If this could be confirmed by further study, guidelines could be adjusted to recommend repeat testing without immediate treatment for those with borderline IGRA results in whom the risk of TB is deemed to be low. Identification of a positive IGRA may have significant implications for the patient, family and the facility. Our experience illustrates the importance of repeating the tests where the results seem anomalous.

Our study had several limitations in addition to its retrospective design. As is common to all studies pertaining to the diagnosis of LTBI, the absence of a reliable gold standard is problematic. Thus, it is difficult to interpret the significance of low positive reverting or converting QFT test results without long term follow-up. A second limitation was that the initial TST testing results were self-read. However this reflects routine clinical practice in many settings with low burden of TB. In addition, our routine was to have the TST repeated and read by a physician or health care worker if the initial QFT was potentially positive. Third, because we restricted retesting to those whose initial results were positive, we were unable to comment on conversion rates. Fourth, the number of children screened was relatively small, limiting the power of our observations. In contrast to findings in adults with CD4 counts < 250 cells/μL [30], we do not think that HIV-related immune suppression was a likely explanation for negative TST or Quantiferon results in our cohort, because the vast majority were healthy and had normal CD4 counts. A relative strength was the validation of our IGRA results by repeat testing in a separate laboratory blinded to our results.

## Conclusion

In conclusion, QFT exhibits a high level of test validity in relatively healthy, immunologically stable HIV infected children. However, many subjects exhibited low level positive test results (0.35 – 1.0 IU/mL), reversion to negative, and lack of correlation with TST results, making interpretation difficult. Future studies are needed to determine the true significance of low positive IGRA results. We recommend repeat testing of low level results, and determination of cut off values at which results remain consistently positive or negative. Guidelines that incorporate QFT use in HIV infected children may need to incorporate the utility of quantitative IFN-gamma values in interpreting QFT results and the need to repeat low positive results.

## Authors’ original submitted files for images

Below are the links to the authors’ original submitted files for images. Authors’ original file for figure 1

## Abbreviations

TB: Tuberculosis
HIV: Human immunodeficiency virus
QFT: Quantiferon Gold-in-tube assay
IGRA: Interferon gamma release assay
TST: Tuberculin skin test
INH: Isoniazid
IFN: Interferon
ART: Anti retroviral therapy.
