# Supplementary figures and images for: Quantiferon Gold-in-tube assay for TB screening in HIV infected children: influence of quantitative values
Source: BMC Infect Dis. 2014 Sep 23;14:516. doi: 10.1186/1471-2334-14-516 (PMC4181619; doi:10.1186/1471-2334-14-516)

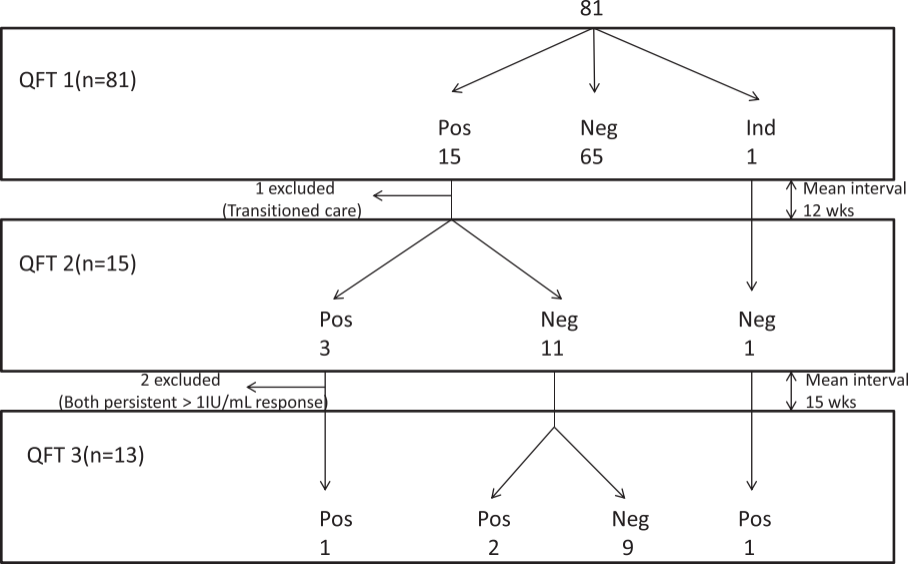

Supplement: Supplementary file 1 — Authors’ original file for figure 1 [file 12879_2014_3834_MOESM1_ESM.pdf]
